# Unchanged Plasma Levels of the Soluble Urokinase Plasminogen Activator Receptor in Elective Coronary Artery Bypass Graft Surgery Patients and Cardiopulmonary Bypass Use

**DOI:** 10.1371/journal.pone.0098923

**Published:** 2014-06-09

**Authors:** Waldemar Gozdzik, Barbara Adamik, Anna Gozdzik, Maciej Rachwalik, Wojciech Kustrzycki, Andrzej Kübler

**Affiliations:** 1 Department of Anesthesiology and Intensive Therapy, Wroclaw Medical University, Wroclaw, Poland; 2 Department of Cardiac Surgery, Wroclaw Medical University, Wroclaw, Poland; University Hospital Medical Centre, Germany

## Abstract

**Objective and Design:**

The soluble urokinase plasminogen activator receptor (suPAR) has been recently recognized as a potential biological marker of various disease states, but the impact of a major surgical intervention on the suPAR level has not yet been established. The aim of our study was to investigate if the induction of a systemic inflammatory reaction in response to cardiopulmonary bypass would be accompanied by an increase in the plasma suPAR level.

**Methods and Subjects:**

Patients undergoing coronary artery bypass grafting under cardiopulmonary bypass (CPB) were added. Based on the baseline suPAR level, patients were divided into group 1 (suPAR within normal range) or group 2 (suPAR above range). Blood was collected before the induction of anesthesia and 6 and 24 hours after surgery. Plasma suPAR, IL-6, IL-8, TNF-α, troponin I, NT-proBNP, and NGAL were quantified to assess the impact of surgical trauma on these markers.

**Results:**

The baseline suPAR level was within the normal range in 31 patients (3.3 ng/mL), and elevated in 29 (5.1 ng/mL) (p<0.001). Baseline mediators of systemic inflammatory reaction concentrations (IL-6, TNF-α, and IL-8) and organ injury indices (troponin I, NT-proBNP, and NGAL) were low and increased after surgery in all patients (p<0.05). The surgery did not cause significant changes in the suPAR level either at 6 or 24 hours after, however the difference between groups observed at baseline remained substantial during the postoperative period.

**Conclusions:**

There was no change in the suPAR level observed in patients subjected to elective cardiac coronary artery bypass surgery and CPB, despite activation of a systemic inflammatory reaction.

## Introduction

Cardiac surgery is known to induce a systemic inflammatory response. The inflammatory mediators are activated and released from blood cells as a result of exposure to the foreign surfaces of the bypass circuit, surgical trauma, hypotension, tissue ischemia-reperfusion, and hypothermia [1], [2]. The signs of a systemic inflammatory response to cardiac surgery with cardiopulmonary bypass (CPB) may be misleading in the diagnosis of postoperative complications that may be the result of infection. The soluble urokinase plasminogen activator receptor (suPAR) has been recognized recently as a potential biological marker of various disease states including cardiovascular disease [3], [4]. An active form of the suPAR molecule is derived from the proteolytic cleavage of the membrane-bound urokinase plasminogen activator receptor (uPAR) [5] and can be detected in different body fluids, including blood, urine, cerebrospinal fluid, and pleural, pericardial, and peritoneal fluids [6], [7], [8], [9], [10]. The soluble form of the uPAR molecule is not subject to further degradation and hence is stable in the blood for 24 hours in healthy people [11]. Numerous studies have shown an elevated level of suPAR in the blood in the course of infectious and inflammatory illnesses [12], [13]. A single determination of suPAR in critically ill ICU patients might be a tool for prognosticating in-hospital mortality on admission to the ICU [14].In addition, changes in the suPAR concentration have been shown to have prognostic value in predicting the outcome of patients with severe sepsis [15]. However, the impact of major surgical intervention on the suPAR level has not yet been established. Because suPAR has been proposed as a biological marker of fibrinolysis and inflammation, we hypothesized that levels of suPAR would be elevated after cardiac surgery and correlate with selective markers of inflammation and organ injury.

## Materials and Methods

### Study design

The study was conducted prospectively in the University Teaching Hospital in Wroclaw, Poland over a period of 6 months. Ethics Committee of Wroclaw Medical University in Poland approved the study protocol. After institutional approval of the study design, written informed consent was obtained from all patients. All clinical investigations were conducted according to the principles expressed in the Declaration of Helsinki. Adult patients undergoing first-time elective coronary artery bypass grafting (CABG) under cardiopulmonary bypass (CPB) were consecutively added to the study. Patients with poor myocardial function (ejection fraction <40%), unstable angina, and other co-morbidities that involve diabetes, renal or liver failure were excluded.

### Intra-operative management

Anaesthesia was induced with propofol and sufentanyl. Pancuronium was given to facilitate intubation. Anaesthesia was maintained with sevoflurane 1–3 Vol%, associated with sufentanyl infusion 0.2–0.5 µg/kg/h. Ventilation was controlled with a mixture of air and oxygen (FiO2 0,6), and the end tidal carbon dioxide concentration was maintained the between 4.7–6.0 kPa. Surgical techniques were the same for all patients and were performed by either of two cardiac surgeons with uniform myocardial protective techniques. In all patients a standard open bypass circuit was used, composed of uncoated polyvinylchloride tubing, a hard-shell venous reservoir, a hollow fiber membrane oxygenator (Sorin Group, Italy) with an integrated polyester arterial line filter of 40 µm pore size, and roller pump with a non-pulsatile flow 2.2–2.4 l/min/m2 (Stockert S3, Sorin Group, Germany) Anticoagulation was attained by administering 300IU/kg of heparin to achieve an activated clotting time longer than 480 seconds. Perfusion pressure was kept at 60 to 80 mmHg. After aortic cross-clamping, intermittent warm blood cardioplegia was delivered in perfusate temperature. Normothermia (37°C) was maintained during the entire procedure. Postoperatively, the patients were transferred to the Intensive Care Unit (ICU). Sedation with propofol 0.5-1.0 mg/kg/h was continued until the patient was able to be weaned from the ventilator and extubated. The postoperative course (24 hours) was monitored to detect myocardial ischemia, the need for inotropic pharmacological support, and heart rhythm disturbances.

### Data collection

The following information was collected from each patient: age, gender, weight, and medications taken before surgery. The ejection fraction and the presence of medical conditions including arterial hypertension, left main stenosis, tripple vessel disease, and previous myocardial infarction were also recorded. The hospital survival rate was recorded and a follow-up was done 6 and 12 months after surgery.

Blood samples to measure suPAR, interleukin 6 (IL-6), interleukin 8 (IL-8), tumor necrosis factor-alpha (TNF-α), Troponin I, N-terminal prohormone of brain natriuretic peptide (NT-proBNP), and neutrophil gelatinase associated lipocalin (NGAL) levels were collected from the arterial blood catheter to the tubes with EDTA as an anticoagulant at the baseline (before the induction of general anesthesia), and 6 and 24 hours after surgery. Blood was centrifuged 0.5 h after collection (3000 rpm for 15 min, +4°C) and plasma samples were stored at −80°C until measurements were performed. The quantitative determination of the soluble suPAR levels was performed with a suPARnostic enzyme-linked immunosorbent assay (ViroGates). It is a commercially available diagnostic kit that has CE/IVD certification. The normal range of values of the plasma suPAR depends on the age and gender of the patient. IL-6, IL-8, and TNF-α were measured with enzyme-linked immunosorbent assay using commercially available kits (BenderMedSystems); NGAL (Quantikine R&D Systems), NT-proBNP (DRG Diagostics). All measurements were done in duplicates with appropriate controls. The results for routine laboratory parameters like troponin I, the c-reactive protein (CRP), aspartate transaminase (AST), alanine transaminase (ALT), creatinine, urea, the white blood cell count (WBC), and platelet count were recorded at baseline, 6 and 24 hours after surgery.

### Statistical analysis

Data were analyzed with Statistica 9.1 (StatSof). The distribution of the variables was not normal based on a Shapiro–Wilk test. Therefore, statistical analysis of the data was performed using nonparametric techniques. Comparisons among different time points were performed by using a Friedman ANOVA by ranks test. The Mann-Whitney U test was used to compare differences between two independent groups. Continuous variables are presented as medians with 25th and 75^th^ percentiles. The relationship between the suPAR level and other parameters was assessed with a Spearman's rank correlation test. Statistical significance was determined as *p*<0.05.

## Results

### Baseline characteristics

60 patients who underwent CABG under CPB were enrolled in the study. Based on the baseline suPAR level, patients were divided into group 1 (N = 31; suPAR within normal range) or group 2 (N = 29; suPAR above range). The median age of the patients was 64 years with a predominance of males (82%). Arterial hypertension, triple vessel disease, and previous myocardial infarction were the most common co-morbidities diagnosed in patients. The median duration of surgery was 233 minutes in group 1 and 241 min in group 2. The majority of patients (65%) needed triple bypass grafts in the procedure. The median CPB duration was 85 min. in group 1 and 79 min. in group 2. Twenty-two (35%) out of 60 patients needed longer time on CPB, *i.e.* >90 minutes, and for those individuals the CPB duration was 105 min. (95–117); for the other 38 patients (CPB time <90 minute) the CPB duration was 76 min. (65–82).

Three patients developed new perioperative myocardial infarction which had been determined by a new Q wave in the electrocardiogram and a rise in the troponin level in the first postoperative day. The length of hospital stay was 9 days (8–11) in group 1, 9 days (8–10) in group 2, and the hospital survival rate was 100%. The mortality of the 60 patients was evaluated after a follow-up of 6 and 12 months. At 6 month follow- up, mortality was 1.6% and it did not change at 12 month follow-up. Baseline characteristics of the study group are presented in Table 1.

**Table 1:** Baseline characteristics of patients.

	Patients with suPAR level within normal range, n = 31	Patients with suPAR level above normal range, n = 29	*p*
**suPAR (ng/mL)**	3.3 (2.7–3.7)	5.1 (4.7–5.5)	0,0001
**Gender F/M**	5/26	6/23	n.s.
**Age (years)**	64 (59–70)	64 (57–73)	n.s.
**Weight (kg)**	80 (70–87)	82 (74–90)	n.s.
**Ejection fraction (%)**	55 (50–60)	57 (55–65)	n.s.
**Co-morbidities n (%)**			
Previous myocardial infarction	24 (77)	22 (76)	
Arterial hypertension	27 (87)	24 (83)	
Left main stenosis	5 (16)	5 (17)	
Triple vessel disease	22 (71)	25 (86)	
**Drugs taken before surgery n (%)**			
Beta blockers	20 (65)	21 (72)	
ACE inhibitors*	17 (55)	21 (72)	
Calcium blockers	10 (32)	5 (17)	
Nitrates	13 (42)	9 (31)	
**Number of grafts** **n (%)**			
2	6 (19)	9 (31)	
3	20 (65)	19 (66)	
4	5 (16)	1 (3)	
**Surgery duration (min)**	233 (210–240)	241 (210–260)	n.s.
**CPB duration (min)**	85 (77–108)	79 (66–94)	n.s.
**Aortic cross clamp time (min)**	45 (37–54)	40 (35–44)	n.s.
**Time to extubation** **(hours)**	8.1 (6.5–8.5)	9.8 (6.5–9.5)	n.s.

*ACE inhibitors, angiotensin-converting-enzyme inhibitor; CPB, cardiopulmonary bypass. Data presented as medians with 25th and 75th percentiles.

### Indices of systemic inflammatory reaction

The baseline value of the CRP was low in both groups and then increased significantly at 24 hours after surgery (p<0.05) and the WBC count was within normal range at all times under observation. The CRP level correlated with the aortic cross clamp time at 24 hours after surgery (R = 0.5, p<0.05). The IL-6 and IL-8 levels were low at baseline then increased at 6 hours after surgery (p<0.0001) in both study groups. At 24 hours after surgery IL-6 and IL-8 values decreased but still remained elevated compared to the baseline (p<0.0001) (table 2). The TNF-α concentration was markedly higher at 6 hours and 24 hours after surgery (p<0.05), compared to the baseline values, in group 2. In group 1, the changes in TNF-α observed after surgery were not significant (p>0.05). No marked differences in CRP, WBC, IL-6, IL-8, TNF-α were noted between groups at baseline and during the postoperative period.

**Table 2:** Indices of systemic inflammatory reaction in group 1(n = 31) and group 2(n = 29), measured at three time intervals.

	Baseline		6 hours after surgery		24 hours after surgery	
	Group 1	Group 2	Group 1	Group 2	Group 1	Group 2
**CRP**	4.0	3.9	not done	not done	106.0*	123*
**(mg/L)**	(3.3–8.0)	(3.1–14)			(76.3–158.7)	(113–171)
**WBC**	9.5	8.6	10.2	10.9	8.7	10.3
**(10^3^/µl)**	(7.4–12.5)	(7.6–9.5)	(7.6–11.4)	(9.1–12.3)	(8.0–11.2)	(9.1–12.4)
**IL-6**	5.1	8.4	84.2#	65.2#	43.0#	52.2#
**(pg/mL)**	(2.2–11.4)	(3.4–12.0)	(61.0–115.0)	(35.3–107.0)	(28.8–75.4)	(29.8–70.8)
**IL-8**	5.9	5,4	35.7#	35.8#	19.3#	19.2#
**(pg/mL)**	(1.9–14.1)	(1.5–12.1)	(26.5–58.7)	(28.3–68.2)	(13.9–24.4)	(14.6–28.5)
**TNFα**	37.4	24.2	50.5	45.1#	41.9	39.3#
**(pg/mL)**	(11.3–46.8)	(10.2–40.3)	(19.7–56.5)	(24.2–80.5)	(19.7–65.5)	(33.7–43.5)

CRP, c-reactive protein; WBC, white blood cell count; IL-6, interleukin 6; IL-8, interleukin 8; TNF alpha, tumor necrosis factor-alpha. Data presented as medians with 25th and 75th percentiles;

*p<0.05 compared to the baseline;

# p<0.0001 compared to the baseline.

### suPAR

**Figure 1:**
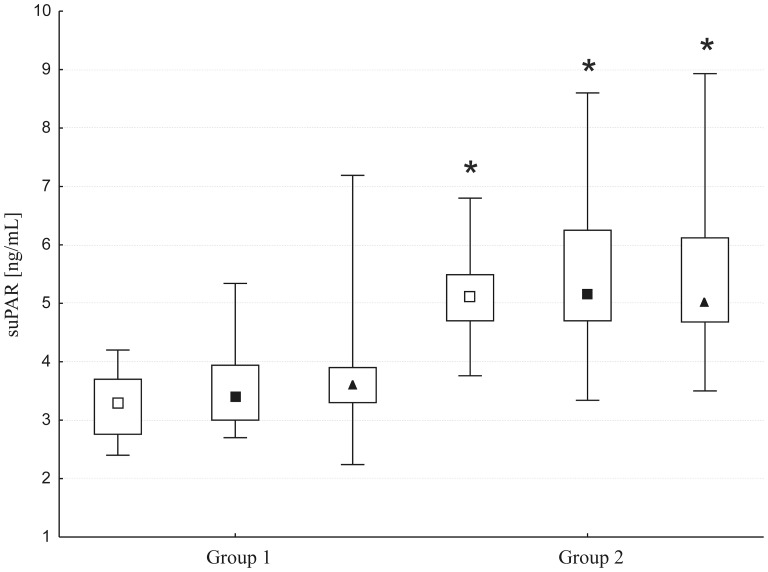
Plasma levels of suPAR measured at baseline (open squares), 6 hours (closed squares), and 24 hours (triangles) after surgery. Data are presented as medians (middle point) with 25th and 75th percentiles (box) and min-max values (whiskers); * p<0.001, a comparison between groups.

SuPAR concentration was measurable in all 180 plasma samples collected from patients. The baseline suPAR level was within the normal range in 52% of patients (group 1, 3.3 ng/mL, 2.7–3.7) and elevated in 48% (group 2, 5.1 ng/mL, 4.7–5.5) (p<0.0001). The surgery did not cause marked changes in the suPAR level either at 6 (group 1: 3.4 ng/mL, 3.0–3.9; group 2: 5.1 ng/mL, 4.7–6.2) or 24 hours after (group 1: 3.7 ng/mL, 3.3–3.9; group 2: 5.0 ng/mL, 4.6–6.1) (Figure 1); however, differences in the suPAR levels in the two groups were significant (p<0.0001) postoperatively. Longer time on CPB (>90 min) was not accompanied by a significant change in the suPAR level at 6 and 24 hours after surgery. The plasma suPAR level correlated with the AST level (r = 0.5, p<0.001) and there was a weak correlation with the NGAL level (r = o.2, p<0.01).

### Organ injury indices

Baseline values of NTproBNP, troponin I, NGAL, creatinine, urea, AST, ALT and platelet count were within normal range in both study groups (Table 3). At 6 hours after surgery there were significant increases in NTproBNP (p<0.05), troponin I (p<0.0001) and NGAL (p<0.0001) in both groups; at the same time we did not see any marked changes in the creatinine, urea, AST and ALT concentrations. At 24 hours after surgery we observed elevated levels of NTproBNP (p<0.0001), troponin I (p<0.0001) and NGAL (p<0.0001); at the same time, we did not see any marked changes in creatinine, urea, and ALT, although the AST level increased (p<0.05) in both groups. The platelet count decreased significantly in both groups at 6 and 24 hours. The troponin I plasma level correlated with the CPB duration and aortic cross clamp time at 6 (R = 0.3, p<0.05) and 24 hours after surgery (R = 0.3, p<0.05).

**Table 3:** Indices of organ damage measured in group 1(n = 32) and group 2(n = 29) measured at three time intervals.

	Baseline		6 hours after surgery		24 hours after surgery	
	Group 1	Group 2	Group 1	Group 2	Group 1	Group 2
**NTproBNP**	0,5	0.4	0.7*	0.6*	37.0#	37.2#
**(pg/mL)**	(0.0–2.3)	(0.0–0.6)	(0.0–5.8)	(0.0–13.7)	(13.0–49.0)	(10.3–76.4)
**Troponin I**	0.02	0.02	6.2#	4.2#	4.4#	2.5#
**(ng/mL)**	(0.01–0.1)	(0.01–0.1)	(3.5–11.9)	(2.8–5.3)	(1.5–8.5)	(1.3–8.4)
**NGAL**	110.7	113.1	159.0#	173.2#	154.7#	130.4*
**(ng/mL)**	(81.8–142.2)	(86.8–138.7)	(128.1–201.3)	(122.2–217.9)	(108.7–183.4)	(101.5–158.2)
**Creatinine**	0.8	0.9	0.8	0.9	0.9	0.9
**(mg/dL)**	(0.7–1.0)	(0.7–1.1)	(0.6–0.9)	(0.8–1.1)	(0.9–0.9)	(0.7–1.1)
**Urea**	32	32	36	32	44	33
**(mg/dL)**	(29–43)	(29–45)	(31–43)	(29–47)	(43–46)	(25–50)
**AST**	22	29	28	35	37*	57*
**(U/L)**	(15–27)	(18–42)	(24–44)	(31–57)	(29–130)	(37–109)
**ALT**	22	35	26	32	28	39
**(U/L)**	(16–41)	(32–52)	(17–40)	(21–41)	(18–51)	(30–55)
**Platelets**	204	244	153*	149#	147*	147#
**[10^3^/µL]**	(188–229)	(184–276)	(145–198)	(113–185)	(134–157)	(123–164)

NT-proBNP, N-terminal prohormone of brain natriuretic peptide; NGAL, neutrophil gelatinase associated lipocalin; AST, *aspartate*.

Data presented as medians with 25th and 75th percentiles;

*p<0.05 compared to the baseline;

# p<0.0001 compared to the baseline.

## Discussion

This is the first study evaluating the effect of a major surgical procedure on the perioperative level of the soluble form of the urokinase plasminogen activator receptor in a population of cardiac surgery patients. Half of the patients requiring bypass surgery in our study group had an elevated suPAR level before surgery and in the others suPAR was within normal range. We demonstrated that cardiac surgery with coronary artery bypass grafting had no impact on changes in the suPAR level in the blood despite concomitant significant activation of an inflammatory response.

Coronary artery bypass grafting activates processes of coagulation and fibrinolysis – integral components of the inflammatory reaction [16]. A number of mechanisms are responsible for the CPB mediated inflammatory response [17], [18]. Cytokines have a major role in the development of the inflammatory reaction caused by surgical trauma. They mediate local inflammatory response to tissue injury and also initiate some of the systemic changes. During CPB, neutrophils are activated primarily by components of both, the complement C3a and C5a fragments and contact systems, and also by the participation of kallikrein and factor XIIf. Other mediators play an influential role as well: thrombin, histamine, and neutrophil-activating peptide 2 released from platelets. They all have been shown to activate neutrophils during CPB. [18]. As a result, selectins expressed on inflamed endothelial cells mediate the first contact between leukocytes and endothelium and initiate leukocyte recruitment. The subsequent activation of neutrophils by a number of pro-inflammatory mediators, including the platelet-activating factor and interleukin-8, provokes an increase in integrin alpha M activation on the leukocyte surface [19]. Released cytokines and chemokines upregulate the expression of the intercellular adhesion molecule (ICAM)-1, the vascular cell adhesion molecule (VCAM)-1, and the platelet-endothelial cell adhesion molecule (PECAM)-1 on the surface of endothelial cells [20]. The binding of integrins to ICAM-1 and VCAM-1 initiates strong adhesion of leukocytes to endothelial cells, leading to their transendothelial migration into the tissue [21]. In our study, a significant increase in the tumor necrosis factor, interleukin-6, and interleukin-8 was observed in the postoperative period, reflecting the activation of the systemic response induced by cardiac surgery. There are a number of studies which have shown a link between suPAR and different markers of inflammation and organ damage [4], [12], [22]. Both membrane-bound and soluble uPARs have been shown to bind to integrins and have been proposed to be involved in cell adhesion and proliferation [23]. The soluble form of uPAR has been reported to have direct chemotactic properties, which may facilitate recruitment of inflammatory cells such as neutrophils and monocytes [24]. In a study published by Koch et al., suPAR concentration measured in 273 ICU patients on admission was independently correlated to TNF-alpha (r = 0.57, p<0.001) and CRP (r = 0.41, p<0.001) level [12], and patients with sepsis demonstrated significantly higher suPAR concentration in comparison to patients without sepsis. In a human model of endotoxemia, suPAR serum concentration increased after *i.v.* administration of a high-dose endotoxin; however, a low-dose of endotoxin modestly increased suPAR release only for the first 1-2 hours, without any systemic effect [25]. Furthermore, in the same model, a 3-hour *i.v.* infusion of pro-inflammatory cytokines, either IL-6 or TNF-alpha, did not have an effect on the suPAR level. Patients in our study did not present signs of infection or clinical symptoms of organ failure after surgery, which may explain the lack of a significant increase in suPAR, despite an elevated cytokine level.

During extracorporeal circulation with CPB a marked increase in the level of polymorphonuclear (PMN) granulocytes elastase was reported as a result of cell activation and the release of elastase from azurophilic granules [26]. In resting neutrophils, uPAR molecules are stored in secretory vesicles and specific azurophilic granules, similarly with elastase, and rapidly translocate upon neutrophil activation to the cell surface [27]. It was previously shown that uPAR expression on immune cells was already elevated before anesthesia in cardiac surgery patients [28]. This phenomenon might reflect ongoing priming and/or activation of immune cells in seriously ill patients requiring cardiac surgery. In the same study, it was reported that the expression of uPAR on granulocytes and monocytes markedly decreased after surgery, which could be explained by uPAR endocytosis at the end of surgery, uPA internalization and rerceptor shedding. Neutrophil-derived suPAR was shown to increase rapidly during neutrophil activation in vitro; TNF-*α*-primed and IL-8-stimulated neutrophils released the chemotactically active suPAR [29]. suPAR is considered to be a strong neutrophil chemoattractant for evaluating the potential mutual dependence between suPAR and neutrophils. The effect of CPB on leukocyte activation was investigated by Roisant et al. [21]. The authors observed that increased leukocyte recruitment during the CPB-induced inflammatory response was caused by an increased chemokine induced arrest and transmigration. We observed that the suPAR level remained stable after surgery. This indicates that surgical trauma itself did not stimulate receptor translocation and release from cell membranes. Also, a longer time of CPB did not affect changes in the suPAR level after surgery. It was previously shown that the expression of membrane-bound uPAR on monocytes and granulocytes remained low after cardiac surgery [28]. The low expression of the membrane-bound receptor observed in that study and no increase in the soluble form of the receptor in the blood in our study suggests that there was no receptor stimulation and further translocation to the cell surface. The clinical relevance of this observation in patients undergoing major surgery warrants further investigation. A potential elevation in suPAR concentrations after major surgery might indicate the need for more detailed monitoring, especially for infectious complications.

To our surprise, the initial level of suPAR was elevated in only 52% of studied patients; almost half of the patients had suPAR within the normal range, despite advanced coronary atherosclerosis, a serious clinical condition requiring cardiac surgery. Apart from the clear link between high suPAR concentrations in the course of infectious diseases, recent studies have pointed to a correlation between suPAR and atherosclerosis [30] and the development of cardiovascular diseases [3], [4], [31], [32]. Low grade inflammation contributes to all stages of atherosclerosis, from the initial phase of increased endothelial permeability to the formation of mature atherosclerotic plaque and plaque rupture [33], [34]. In Lyngbaek's study of 296 patients with ST-segment elevation myocardial infarction (STEMI) treated with primary percutaneous intervention (PCI), the baseline suPAR level was low in 25% of patients and only slightly elevated in another 25% [35]. Additionally, the level of suPAR remained stable for 25 hours from the onset of STEMI. A similar result was observed in our study: there was no correlation of suPAR with the troponin I level noted at any point in time, regardless of patients' clinical condition, heart rhythm disturbances or the need for inotropic pharmacological support after surgery. However, despite the absence of major adverse cardiac events during the perioperative course in our study group, a significant increase in several organ injury indices was recorded after surgery. We observed that markers of heart failure, NT-proBNP and troponin I, were higher postoperatively. Recently, Sammy et al. have shown that levels of Nt-proBNP significantly increased in patients who developed organ dysfunction on the first postoperative day [36]. A threshold value of Nt-proBNP of 307 pg/mL at 4 hours after CPB correlates to the occurrence of arrhythmia after cardiac surgery with CPB with a sensitivity of 71% and a specificity of 84%. The values of Nt-proBNP recorded in our study were much lower: 19.8±77.5 pg/mL and 48.9±51.9 pg/mL at 6 and 24 hours after the surgery, and heart rhythm disturbances were not observed.

A decrease in the platelet count was observed, although there were no noted problems with bleeding after surgery. Low platelet count during CPB surgery was previously described [37] along with a compensating increase in platelet activity.

From all measured indices of organ injury, the NGAL level and AST activity correlated with the soluble uPAR level. NGAL is a sensitive, early indicator of renal tubular damage. It participates in the modulation of inflammation and degradation of the extracellular matrix and is considered to be an acute phase protein [38], [39]. NGAL can be activated on smooth muscle cells and atherosclerotic plaque [40]. It was previously shown that in patients with acute heart failure after myocardial infarction, as well as with chronic heart failure regardless of etiology, an elevated level of NGAL in serum correlated with a worsening of the clinical condition of the patients [41].

We are aware that our study has several limitations resulting from the size of the study group. Another limitation is the inability to assess the usefulness of plasma suPAR concentrations as a diagnostic and prognostic indicator of the development and intensity of serious inflammatory response complications, including sepsis and organ failure after CABG surgery with CPB. As our patients were a low risk population and both perioperative and postoperative courses were mostly uneventful, it is not possible to extrapolate our findings to higher risk patients.

Further studies are warranted to reveal whether changes in suPAR expression correlate with the severity of the inflammatory response in higher risk cardiac surgical patients. suPAR is implicated in the inflammatory response, but whether it induces the immune response or whether changes in suPAR are just a result of the increased activation of the immune system is still not clear.

In summary, we observed that the suPAR level did not change as a result of surgical stress and the activation of the immune system. It should be emphasized that 48% of studied patients had a baseline suPAR level within the normal range, despite a very serious clinical condition and the diagnosis of severe heart failure requiring coronary artery bypass graft surgery. The level of suPAR correlated with the rate of NGAL concentration and additionally appeared to be related to liver function. As presented here, the experimental model of inducing an inflammatory response in the course of surgery could serve as a model for future studies involving surgery and suPAR. An increase in the plasma suPAR level after major surgery might indicate the need for more detailed monitoring, especially for infectious complications; however, larger clinical studies are required in order to understand the regulatory mechanisms of the release of suPAR.
